# Retraction: Ubiquitination increases Parkin activity to promote Autophagic α-Synuclein Clearance

**DOI:** 10.1371/journal.pone.0324473

**Published:** 2025-05-12

**Authors:** The PLOS One Editors

Following the publication of this article [1], concerns were raised regarding results presented in Figs 1, 2, 3, and 4. Specifically,

- The following panels or parts of panels appear similar despite being used to represent different experimental conditions:
  - Fig 1F of this article [1] and Fig 1M* of [2].
  - Figs 1G and 1J of this article [1], and Supl. Figs 1A and 1D, respectively, of [3].
  - Figs 1H, 1I, 1K, and 1L of this article [1], and Supl. Figs 1B, 1C, 1E, and 1F, respectively, of [3].
  - Fig 2K actin panel lanes 1-5 and Fig 4V Syn panel lanes 3-7 when flipped horizontally.
  - Fig 3C LC3-B panel of [1] and Fig 1G Tau panel of [3] when rotated.
  - Figs 4J, 4K, and 4L of this article [1], and Fig 5B of [4].
  - Fig 4R and Fig 4S.
- In the Fig 2K IP: Ub, WB:parkin panel, there appears to be a vertical irregularity suggestive of image splicing between lanes 5 and 6.

Regarding the panel overlap concerns in the immunostaining results presented in Figs 1 and 4, the corresponding author stated that the figures in this article were prepared using a figure template and that errors were made during the updating of the templates. They provided updated panels and underlying data for editorial review. Upon editorial review of the underlying data, multiple additional instances of full and partial panel similarities were detected for data presenting different experimental conditions.

Regarding the concerns with overlap between gel and blot results, for Fig 2K the corresponding author confirmed that the results in the Fig 2K IP: Ub, WB:parkin panel were prepared using spliced results, and stated that the actin blot was included in error. No underlying data for the blots in Figure 2K and 4V were provided. For Fig 3C, the corresponding author stated that the LC3-B and Tubulin panels were included in error and should not have been presented in this article. Editorial assessment of the underlying data provided for the Fig 3F α-Synuclein results suggests that the protein sizes have not been labeled accurately in [1].

In light of the above concerns that call into question the reliability and integrity of the published Fig 1-4 results and the associated underlying data, the *PLOS One* Editors retract this article.

CEHM agreed with the retraction and apologized for the issues with the article. IL, NMD, and MLH either did not respond directly or could not be reached.

Fig 1F of this article appears to report material from [2] published in 2013 by John Wiley and Sons, Ltd, which is offered under a CC BY license but the original article was not attributed in [1] in accordance with the CC BY license requirements. In addition, the Fig 4J, 4K, and 4L panels appear to report material from [4], published in 2013 by Oxford University Press, which is not available under a CC BY license. Due to restrictions that apply to the original article’s license, the Fig 4J, 4K, and 4L panels are excluded from the PLOS article’s [1] CC BY 4.0 license. The article [1] was republished on April 25, 2025, to note this exclusion in the Fig 4 legend and the article’s copyright statement, and to include appropriate attribution in the figure legend of Fig 1.
